# Intraoperative Gastric Tube Intubation: A Summary of Case Studies and Review of the Literature

**DOI:** 10.4236/ojanes.2017.73005

**Published:** 2017-03

**Authors:** Michael Long, Melissa Machan, Luis Tollinche

**Affiliations:** 1 Department of Anesthesiology and Critical Care Medicine, Memorial Sloan Kettering Cancer Center, New York, USA; 2 Barry University, College of Health Sciences, Hollywood, USA; 3 Department of Anesthesiology, Plantation General Hospital, Plantation, USA

**Keywords:** Nasogastric Tube, Orogastric Tube, Gastric Tube, Perioperative, Intraoperative, Anesthesia, Blind Insertion

## Abstract

**Study Objective:**

Establish complications and risk factors that are associated with blind tube insertion, evaluate the validity of correct placement verification methods, establish the rationales supporting its employment by anesthesia providers, and describe various deployment facilitators described in current literature.

**Measurements:**

An exhaustive literature review of the databases Medline, CINAHL, Cochrane Collaboration, Scopus, and Google Scholar was performed applying the search terms “gastric tube”, “complications”, “decompression”, “blind insertion”, “perioperative”, “intraoperative” in various order sequences. A five-year limit was applied to limit the number and timeliness of articles selected.

**Main Results:**

Patients are exposed to potentially serious morbidity and mortality from blindly inserted gastric tubes. Risk factors associated with malposition include blind insertion, the presence of endotracheal tubes, altered sensorium, and previous tube misplacements. Pulmonary aspiration risk prevention remains the only indication for anesthesia-related intraoperative use. There are no singularly effective tools that predict or verify the proper placement of blindly inserted gastric tubes. Current placement facilitation techniques are perpetuated through anecdotal experience and technique variability warrants further study.

**Conclusion:**

In the absence of aspiration risk factors or the need for surgical decompression in ASA classification I & II patients, a moratorium should be instituted on the elective use of gastric tubes.

## 1. Introduction

According to a healthcare study published by the Society of Actuaries in 2010, over 19 billion healthcare dollars were spent in the United States on preventable medical errors [1]. Iatrogenic injury caused by blind gastric tube insertion is one such error that carries high morbidity and mortality. In 2013, over a million nasogastric tubes were inserted in the United States and the reported rate of misplacements in medical literature was estimated at 1.2% – 2.4% with over half occurring in mechanically ventilated patients [2] [3]. Anesthesia providers routinely perform blind intubations in this vulnerable population and cite surgical and anesthetic indications as rationales for placement during general endotracheal anesthesia (GETA). However, given the blind nature of this technique, patients are subject to adverse sequelae from a commonly performed and familiar treatment option.

The sump drainage tube is the most often deployed device during the perioperative period and features a dual lumen design. One lumen allows for drainage or as a conduit for medications and lavage while the second lumen functions to permit air passage. Lacking this innovation, negative pressure can result in a stomach lining injury or promote a mechanical obstruction. This design helps mitigate this risk and is intended to prevent the mucosal wall from refluxing back into the tubing. The dual lumen sump drainage tube is often inserted in emergency room patients requiring emergent gastric decompression or lavage and is used regularly in the perioperative and postoperative management of surgical patients. When used intraoperatively, the gastric tube is used to evacuate air or fluid retained in the stomach. Gastric tubes are often placed in patients undergoing general anesthesia even in the absence of a surgical indication. Those supporting its practice believe that routine gastric content decompression can ameliorate postoperative nausea and vomiting (PONV). Other providers maintain that this practice reduces aspiration risk by evacuating gastric contents that are produced throughout the intraoperative period. However, literature advocating gastric decompression by anesthesia providers is largely deficient. This was compellingly illustrated in an editorial aptly named “The other tube in the airway: what do we know about it?” by Martin & Aunspaugh [4]. They maintain that gastric tube use by anesthesia providers is based on ritual and that evidential support is lacking. Additionally, they called for more research and dialogue to examine the value of gastric tube insertions in the absence of aspiration risk or surgical indications.

The purpose of this literature review is to establish complications and risk factors that are associated with blind tube insertion, evaluate the validity of correct placement verification methods, establish the rationales supporting its employment by anesthesia providers, and describe various deployment facilitators described in current literature. Due to the multitude of terms that have resulted from manufacturer branding, the tube’s primary purpose, and terminal tip location, “gastric tube (GT)” will be applied throughout the remainder of this review. It will be generically applied to any artificial enteric device inserted orally or nasally to decompress, lavage, and/or administer medication and nutrients to the alimentary tract.

## 2. Methods

To maintain validity and for the purpose of disseminating the most current evidence directed at this review’s objective, a five year limit was applied to exclude any literature published prior to 2009. Pre-search inclusion criteria were literature reviews, randomized controlled trials, and cohort studies in the English language (or those foreign articles conveniently translated into English.) All subject age groups were included for review since the practice of gastric tube insertion, regardless of age, lies within an anesthesia provider’s scope of practice. Exclusion criteria included literature that described a singular case study or literature reviewing case studies and articles whose primary purpose was related to the goal of perioperative gastric decompression.

**Figure 1:**
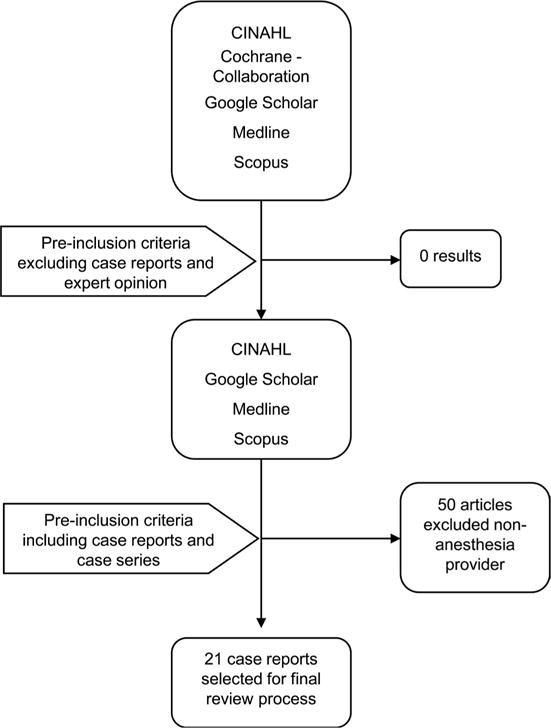
Search strategy.

An exhaustive literature review of the databases Medline, CINAHL, Cochrane Collaboration, Scopus and Google Scholar was performed applying the search terms “gastric tube”, “complications”, “decompression”, “blind insertion”, “perioperative” and “intraoperative” in various order sequences. The various orders sequences employed were gastric tube + complications; gastric tube + decompression; gastric tube + perioperative; gastric tube + intraoperative; gastric tube + blind insertion. This literature search strategy revealed no review articles or prospective/retrospective investigation of any kind related to complications associated with blind GT intubations by anesthesia professionals during the perioperative period. However, evidenced by the amount of complication case reports and GT placement facilitation methods published in anesthesia and surgical peer-reviewed journals, one would surmise that a problem does exist and warrants further investigation. The described five year limitation and data extraction measures were applied. Twenty-one case studies were selected for this review after they were carefully scrutinized to make certain that those involved in the GT insertion or management of the affected patient were anesthesia professionals (see Figure 1). Due to the paucity of literature pertaining to GT complication incidence among anesthesia professionals a short review of medical literature is reasonable. It is not this review’s intent to provide a similarity comparison, but to illuminate the unfavorable sequelae that can potentially occur as the result of the blind method of insertion used by other health professionals engaging in this practice. All other articles in this review were restricted to a five year limit as well for the purposes of disseminating only the most current evidence.

## 3. Results

### 3.1. Complications and Risk Factors

#### Case Reports

Invasive medical procedures convey some level of risk; however, it is the blind unassisted method of insertion that gives the GT the potential for serious harm. The ipsilateral piriform sinus and arytenoid cartilages are recognized as the most common points of resistance as the tube enters the hypopharynx [5]. Aggressive tube handling and the GT’s predilection for low resistant tracks may also be factors that contribute to the complications seen with blind intubations. Due to the early anatomically shared respiratory and alimentary structural pathway, tubes can enter the tracheo-broncho-pulmonary system and be the cause of significant injury. Moreover, malposition may be underappreciated due to the removal of misplaced tubes prior to incurring injury and the provider’s ignorance of the incorrect position [3]. Twenty-one case reports describe complications or iatrogenic injury as the result of blind GT intubation by anesthesia professionals (see Table 1).

In the absence of gastric aspirate or with the suspicion of malposition several case reports have identified intrapulmonary misplacement precluding injury. This type of malposition can occur due to the shared anatomic pathway and the close proximity of the glottis in relationship to the opening of the esophagus. Govindarajulu *et al.* identified an endobronchial placed GT by direct laryngoscopy (DL) subsequent to a ventilator leak [6]. Similarly, Sahu & Baliarsing report a reservoir bag collapse caused by a tracheal misplaced GT even after a false positive when employing the epigastric air auscultation method for verification [7]. Both misplacements were identified immediately and removed prior to incurring injury. Kavala *et al.* reported an uncomplicated GT insertion, not recognized intra-operatively, that was endobronchially malpositioned diagnosed by chest x-ray in the post-anesthesia care unit [8]. The authors recalled difficulties with maintaining ventilation however were unable to discern the causative factor. Ventilator failure, leak or difficulty in delivering ventilation should alert the practitioner to a possibility of pulmonary GT malposition. However, these ventilatory cues resulting from the negative pressure caused by the application of suctioning are not always present or diagnostic of an endobronchial placement and can go unrecognized. The potential harm introduced by an unrecognized pulmonary malposition is described in 3 case reports by Raut *et al*, Kerforne *et al*, and Nanjegowda *et al.* [9] [10] [11]. They all report missed bronchial malpositioned GTs even in the absence of the difficulties depicted by the prior case studies. Kerforne *et al.* reported a methylene blue injection into the GT and upon noting dye present inside the endotracheal tube (ETT) realized the pulmonary malposition. Nanjegowda *et al.* describe refractory laryngospasm in a patient with an endobronchial malpositioned GT that persisted until the offending object was removed. Hegde & Rao, described as a “near miss,” recount events preceding the patient’s ultimate demise that included a bronchially malposition GT in an already respiratorily compromised patient [12]. A GT placed into the lung emphasizes the importance of verification procedures after insertions that are specific in identifying pulmonary GT misplacements. However, even the gold standard for GT placement verification retains its own limitations which are related to errors in human interpretation. Khanna *et al.* presented a case where the gastric tube position was indeterminate. Even a confirmation by radiograph proved inconclusive; giving the appearance of thoracic malposition when in fact the tube was correctly positioned [13].

Complications are sometimes related to the mechanics of the tube itself and even correctly placed tubes can create a quandary or contribute to an injury. Tube coiling and knotting were described in 4 case studies with 2 GTs wrapping around endotracheal tubes (ETTs), one self-knotting through a supraglottic device and an intranasal temperature probe/GT entanglement. Acharya *et al*, Chaudhary *et al*, and Garg & Kapoor all recount intraoperative events where the GT became entangled around other insitu devices or self knotted preventing unhindered GT removal [13] [14] [15]. The entanglements occurred with an endotracheal tube, a nasopharyngeal temperature probe and an supraglottic airway, respectively. Complications were avoided by carefully removing the GT and offending object en masse with particular attention to avoiding injury. However, if unrecognized a traumatic soft tissue injury is reasonable especially if the removal is through orifices that may not permit the easy extraction of a knotted mass. In one ETT/GT entanglement reported by Lin *et al*, the coiling of the GT around the ETT was constrictive enough to cause negative pressure pulmonary edema [16]. The presenting signs were high peak airway pressures, decrease in tidal volumes, and oxygen desaturation to 85%. The diagnosis was confirmed with direct laryngoscopy after bronchospasm and endobronchial intubation were ruled out.

Mucosal or soft tissue trauma is not always avoidable and is primarily due to the blind nature of the insertion. Without the ability to visualize the entire placement and the presence of fragile and often friable structures lying in the path of the GT, blind insertions can contribute to serious harm. Traumatic injury resulting in superficial damage, perforation or fistula formation was reported in 6 case studies and either urgent or emergent procedures ensued as a result of this complication. Burad *et al.* [17] and Hirshoren *et al.* [18] both reported serious soft tissue injuries prompting intervention and subsequent intensive care evaluation post-procedure. Gastric perforation was described by Daliya *et al.* [19] and Hynh *et al.* [20]; both resulted in diagnostic re-operations and consequent surgical repair. Other high morbidity complications, including esophageal perforation, were identified as well. A case report by Turabi *et al.* had recounted multiple difficult unsuccessful GT insertion attempts that resulted in pneumothorax and an esophageal stent for the treatment of the iatrogenic tear [21]. Isik *et al.* recounted an esophageal perforation from a perioperative GT insertion on post-operative day two [22]. The perforation was diagnosed by esophagogastroduodenoscopy (EGD) and the patient underwent an emergent thoracotomy and jejunostomy placement. This highlights the susceptibility of injury from a blindly inserted GT even without reported placement difficulty.

Gastric tubes should be inspected after removal in the same fashion as one would a central line or other invasive tube. A fractured portion of the GT was coughed up by a patient in the post anesthesia care unit reported by Ranier & Costello [23]. Unrecognized intraoperatively, the GT was found to be fractured at the terminal end when retrieved for inspection. The gastric tube featured an inner lumen strikingly similar to the outer lumen which gave the appearance of an intact tube upon removal. This scenario emphasizes the need for practitioners to familiarize themselves with institution specific products and the importance of vigilance during extraction procedures.

Resistance or inability to advance the GT should alert the practitioner to a possible difficult placement. Many of the reports described resistance or difficulty in the primary placement while others depicted more than one or multiple attempts. Resistance encountered should therefore prompt the practitioner to abort any more attempts and instead use alternate methods of insertion especially with a patient specific condition that would predispose them to injurious sequelae. Ching *et al.* describe a case in which after meeting resistance avoided further re-insertions to prevent an injury in a post-esophagogastrectomy patient [24]. This allowed surgical involvement in the placement of the GT which likely avoided a mucosal injury in a susceptible patient.

#### Complication & Risk Factors —Medical Literature

Malpositioning was identified as the most common complication [25] [26] [27] [28] [29] associated with blind GT intubation. This complication is often underappreciated and un-recognized by the provider due to the lack of resistance encountered [30] and subsequent removal of the malpositioned tube prior to incurring injury [2].

Thoracic injuries were identified in five of the seven complication articles with pneumothorax cited as one of the severe adverse sequelae that resulted in death [3] [25] [26] [28] [29]. A systematic review of 9931 total patients, including data derived from four randomized controlled trials (RCT) and one case controlled study, reported a 2% rate of tracheobronchial malpositioning [25]. A literature review in 2014, consisting of a large heterogeneous cohort of critically ill patients from five research studies, conferred a significant association between pulmonary trauma and high morbidity and mortality rates [28]. However, underreporting and ignorance of tube malposition may contribute to lower rates [29] and therefore the prevalence of injury is likely underappreciated. Along with malpositioning, morbidity and mortality associated with broncho-pulmonary injury should be of concern and those performing blind GT insertions must recognize its potential for harm.

Other sites for misplacements are associated with overzealous handling, unintentional tube coiling, kinking of the tube within the alimentary tract, or inadequate length insertion. Esophageal placements due to dislodgement or tube coiling are other areas where malpositioning can occur. Esophageal placement increases aspiration risk [25] [27], contributes to symptoms of gastro-esophageal reflux [27] and results in perforation with or without pneumothorax [26] [29]. This is particularly important especially due to the high mortality rate (13.2%) associated with iatrogenic esophageal perforation and was substantiated by a meta-analysis in 2013 ([31]. A descriptive study, reviewing 381 consecutive radiographs in neonates over a 1-year period, reported a misplacement rate of 59% of which 6% were located in the esophagus [27]. The investigators report a low rate of broncho-pulmonary misplacements in the neonate population. However, study design, one-plane radiographs, and the lack of routine radiographs may have limited their conclusions.

#### Risk Factors

Certain predisposing risk factors have been attributed to blind GT malpositioning; factors of which every practitioner engaging in this particular practice should be cognizant. Sparks *et al.* found mechanical ventilation a factor in 113/187 (60.4%) misplacements with no difference with respect to ventilator setting or presence of ETT or tracheostomy [25]. This was corroborated by another review in 2010, by Giantsou & Gunning [29], citing it as a factor in more than 50% of misplacements. Of these, 66% developed serious thoracic complications such as pneumothorax (80%). A cuffed ETT offered no protection [28] or unexpectedly contributed to an increased risk for broncho-pulmonary displacement [3]. Quandt *et al.* [27] also found an association between the presence of an ETT and lower esophageal misplacement in neonates (odds ratio 2.74, 95% Confidence Interval, *P* = 0.003). In 2014, data collected from 1 RCT and 7 observational studies identified younger age, experience of provider, depressed gag reflex, altered level of consciousness and tube type/size factors associated with misplacements in children [26]. The previously mentioned risk factors, in addition to critical illness and abdominal distention, were also implicated with malpositioning in adults [28]. The choice of endotracheal cuff can also affect malpositioning rates. The introduction of high volume low-pressure cuffs ETTs are believed to be a risk factor due to the softer cuff texture compared to previously utilized low volume high-pressure cuffs [29]. Two literature reviews identified prior unsuccessful GT attempts as another factor contributing to the malpositioned tubes [25] [31]. Sparks *et al.* cited repeat misplacement rates following tube repositioning as high as 32% [25]. They calculated a cumulative mortality rate greater than 20% with repeat attempts. This propensity for increased morbidity caused by repeat attempts at insertion was corroborated by Marderstein *et al.* [31] who found that of those with one previous misplaced tube, 32% resulted in multiple repeated misplacements which increased the risk for pneumothorax with each additional malposition (p < 0.05). Before attempting a secondary blind insertion one should consider the risk exposure when deciding whether that risk supersedes the previously perceived benefits.

### 3.2. Verification Methods

Radiographs remain the standard to which all other verification methods are compared when testing for accuracy. Although the gold standard, the routine use of radiographs as a verification technique is limited by cost, time constraints and is subject to incorrect interpretation. Misinterpretation of radiographs was highlighted in a 6-month prospective re-audit that identified a 17% rate of reading errors by radiologists [32]. All errors were related to unrecognized tube placement errors including a right main bronchus GT intubation. However, during the intraoperative period GT are placed blindly and most never verified by radiography for practical reasons.

Several portable verification methods are currently available for use and are extensively discussed throughout nursing and medical literature. Capnometry or capnography, biochemical testing, and ultrasonography are all methods available for use in the operating room. These techniques carry significant advantage over radiographs in their portability and ready availability of equipment.

#### Unsupported Methods

The auscultation method involves the instillation of air while simultaneously listening over the epigastrium for noise caused by turbulent airflow entering the stomach. Air instillation, even with broncho-pulmonary or esophageal misplacement, can elicit a noise similar to that heard with correct gastric placement and often mistakenly results in a false prediction assessment [33] [34] [35] [36] [37]. Error rates as high as 50% were noted by Makic *et al.* [37] and proved to be unreliable or ineffective with most suggesting its abolishment as a verification method [34] [35] [37] [38] [39]. Despite a wealth of literature spanning 20 years calling into question its validity and practice alerts made by major associations, two systematic reviews identified it as the most widely used verification method among nurses [33] [34]. Parenthetically, four of the GT complication case reports [7] [11] [12] [13] reviewed used the auscultation technique to verify placement. Nanjegowda [11] *et al.* and Sahu & Baliarsing [7] both reported air auscultated over the epigastric although the GT was veritably endobronchial.

Tube aspirate can be obtained to visually distinguish between intestinal and GT placements. However, due to color and consistency similarities shared by gastric and pulmonary aspirates, this method is unreliable with broncho-pulmonary misplacements [33] [34] [37] [38]. Supported by a wealth of evidence, both techniques should be abolished as predictive methods used to verify terminal tip location.

#### Aspirate and Biochemical Markers

Bilirubin, pH, and pepsin/trypsin are biochemical markers that can be identified by testing fluid aspirated from the GT and used in the prediction of terminal tip location. A systematic review, with stringent inclusion criteria and quality assessments, determined that pH testing alone was unreliable [40]. Moreover, the authors found that when coupled with bilirubin, this resulted in a high specificity rate (0.99) for intestinal tube position but was poorly sensitive in identifying stomach tube placements. Conclusively, the investigators were unable to support the accuracy of such methods and suggested the need for stronger evidence. Tests with higher pH value thresholds proved inconsistent when used alone [37] [39] [40] and were limited by commonly prescribed drugs that raise stomach pH [33] [35]. Only one study established pH to be an effective tool when determining tube position, but was limited by small sample size and the objectionable use of pH paper in lieu of pH meters [39]. Ultimately, successful biochemical testing is wholly dependent on the ability to obtain aspirate fluid and tube collapsibility was cited as a frequent obstacle to obtaining a sample for testing [40].

#### Capnometry /Capnography

Carbon dioxide (CO_2_) detection is a verification method readily available to anesthesia providers. Capnometry and capnography are fundamentally similar except capnography provides a continuous analysis of CO_2_ by waveform and capnometry is conceptually a point of care modality. The strongest level of evidence pertaining to CO_2_ as a verification tool is a meta-analysis that supports the use of capnography/capnometry [41]. However, the authors maintain that its role is limited to broncho-pulmonary misplacement identification and not effective in alimentary tract malposition recognition. Turgay & Khorshid [39] also supported this method’s validity for assessing broncho-pulmonary malpositioning, but advised that this must also be combined with biochemical markers to detect esophageal placements. Another factor that can limit its usefulness in detecting pulmonary misplacement is the presence of luminal fluid blocking the flow of gas mixtures needed for CO_2_ detection to occur [35]. Other limitations to the previously reviewed techniques are costs and additional resource allocation imposed by point-of-care testing and a periodic competency evaluation requirement [33].

#### Ultrasonography

Ultrasonography machines, found in almost every anesthesia department, provide a non-invasive and radiation-free modality that decreases body fluid borne contagion exposure. Five publications discussed ultrasonography were selected for review of which three (all cohort studies) supported its use as a predictive measure [34] [42] [43] [44] [45]. However, one RCT, deficient in power, identified low specificity predictability and conceded its use should only be considered a possibility [45]. The only article negating its usefulness was a review article that assessed literature which predated this review’s ultrasound publications [34]. Therefore, prior to validation as a verification tool, larger multi-institutional clinical trials are needed to account for confounding variables like ultrasound handler experience, air artifact that may confuse terminal tip location, and population sample homogeneity.

### 3.3. Correct Placement Facilitating Methods

This is a problem plaguing providers and has produced unique correct GT placement methodologies dating back to the 1960s [46]. However, most reported techniques are novel and anecdotal in their approach, require specialized equipment and/or are accompanied by instructions that are often mired in step-by-step complexities that would discourage adoption. Regardless of the approach or method, some practices have been described in the literature attesting to significant clinical success as a practice that facilitates correct GT deployment.

#### Neck manipulative techniques

Neck flexion, cited by two RCTs, with/ without lateral pressure reported an 88% - 92% success rate [47] [48]. Yet this rate was challenged by a larger RCT by Kirtania *et al*, which used neck flexion/lateral pressure as the comparative analysis control group [49]. The author reported a 56.7% first attempt success rate in the neck flexion/lateral pressure control group as opposed to a 92% rate of success in the experimental cohort (p < 0.001). Lifting of the larynx, another manipulative technique, was cited as having a 92% success rate [49]. However, the described clinical trials were limited by using experienced anesthesia providers and lacking established standardized processes with each described technique.

#### Facilitators requiring specialized equipment or tube modifications

Several articles depict methods that employ specialized equipment with or without tube modifications or replacements. The Glidescope and King Vision video laryngoscopes corroborated higher success rates when used to facilitate placement compared to controls [50] [51]. Despite encouraging success rates, having to re-instrument the oropharynx due to first attempt failures is not without its own attendant risks. In addition, improved rates were attributed to the Rusch stylet and various guidewires by providing a path that avoided areas of resistance [48] [49] [52]. Although successful, alimentary tract malpositioning remains a possibility and guidewire reinforced tubes have been associated with traumatic pulmonary complications. Moon *et al.* [53] attest to the increase in placement success, but also caution against placement guidance without the aid of an ultrasound. Another technique suggested a frozen GT to ease placement with distilled water used as the reinforcement medium to maintain the original coiled shape [54]. The authors reported a correct placement rate (88%) that was significantly higher than the control group. However, they acknowledged that the melting water, due to body temperature, places the patient at risk for pulmonary aspiration if pulmonary intubation should occur.

Ultimately, the conclusions of any previously described employment methods are severely limited by lack of trial reproduction in current literature and their anecdotal nature. In addition, all described methods are limited by the absence of technique standardization and the potential exclusion of subjects that would provide a more representative patient sample. Although these techniques require a dataset with larger, more methodologically sound clinical trials for complete endorsement as placement facilitators most of the described facilitation methods were significantly associated with higher rates of correct GT placement. Therefore, it would be prudent to consider adopting a method that suits your specific skillset given the cumulative morbidity risk exposure of repeat attempts.

### 3.4. Employment Rationales

A myriad of unsupported rationales for the intraoperative use of GT can be accredited to the perpetuation of misinformation that has been instrumental in its continued practice. The only described rationales, discussed in peer-reviewed literature, are for PONV and aspiration risk prevention. Any other rationale professed by anesthesia providers for its intraoperative employment is spurious and unsupported.

#### PONV Prevention

Postoperative nausea and vomiting is the most common anesthesia side effect in the postoperative period with rates occurring up to 70% in certain high risk patients [54]. Postoperative nausea and vomiting can result in prolonged hospital admissions and contribute significantly to healthcare expenditures [55]. Known risk factors include non-smoking females, previous history of PONV or motion sickness, volatile agents, nitrous oxide, length of surgery and intraoperative opioid administration [56]. In attempts to decrease PONV, non-pharmacological methods have been employed by anesthesia providers, with gastric decompression being the most invasive.

Four out of the six articles pertaining to PONV found decompression by GT to be ineffective. Three RCTs conducted on pediatric populations, all reported no statistical significance when compared to the control group [57] [58] [59]. However, they were limited by small sample sizes [57] [58] and Al-Khtoum *et al.* [59] did not provide the statistical methodology used when reporting a higher PONV rate in the control and oro-GT groups. The study with the largest sample size involving adults was the only non-RCT meeting the inclusion/exclusion criteria assessing the effectiveness of PONV [60]. Using data from a previously published clinical trial, Kerger *et al.* used propensity scoring, identifying matched pairs, to mimic randomization in their observational study. They reported no evidence supporting the use of intraoperative or perioperative GTs (p = 0.35 and p = 0.61 respectively) for PONV purposes.

Those finding gastric decompression either effective or reporting a significant change when compared to controls were both RCTs on adult patients undergoing various cardiac and ENT surgeries. Lavi *et al*, in 2011, randomized 202 patients undergoing cardiac surgery, and found vomiting significantly higher in the non-GT group [61]. They also noted that the severity of vomiting was related to the amount of aspirate volume but found no difference in nausea between the groups. Having more smokers in the control group strengthened their conclusions, however they did not institute controls on volatile agent usage by anesthesia providers. This may be considered a weakness affecting the study’s outcomes since volatile agents are a known PONV risk factor. The clinical study by Erkalp *et al*, a recent multicenter study in patients undergoing ENT surgeries, found it to be beneficial and advocated its use in all patients undergoing ENT surgeries [62]. They found the severity of PONV to be significant between the groups, in the 2^nd^ hour of recovery and up to 24 hours after surgery. However, limitations to their trial were related to the small sample size and profound heterogeneity of ENT cases accepted for enrollment.

Currently, the evidence is lacking and what is available is more questionable than supportive. The new consensus guidelines adopted by the American Academy of Anesthesiologist Assistants, the American Association of Nurse Anesthetists and the American Society of Anesthesiologists have removed GT use for PONV prevention citing Kerger *et al.* [60] as the basis for the revision [56].

#### Aspiration risk prevention

Gastric tubes for aspiration risk prevention are supported by a recent review article by Salem *et al.* [63]. This led to the subsequent development of an algorithmic tool that assists anesthesia providers in the risk stratification process based on predisposing risk factors and patient acuity. Furthermore, the authors acknowledged the lack of sufficient clinical data pertaining to GT utilization in those deemed high risk for pulmonary aspiration. Designing a clinical trial to evaluate its efficacy in decreasing aspiration risk poses many challenges. Despite the lack of evidence, this practice will remain as a result of the ethical considerations and gravity associated with pulmonary aspiration.

### 3.5. Limitations & Gaps in the Literature

The main limitation to this review is the lack of complication-related articles that specifically pertain to GTs inserted by anesthesia providers. More prospective observational studies need to be performed to identify the incidence of GT malposition during the intraoperative period, the effectiveness of intraoperative gastric decompression and whether current verification methods are practical in the perioperative setting. Although medical literature suggests complication rates as high as 16%, too many differences exist between surgical and medical patients. These variables, which may include differences in population, use of muscle relaxants, qualification of the practitioner, and type of tube inserted, may not extrapolate to the intraoperative setting. Yet, similar risk factors are present between the two that warrant attention like the presence of an ETT and patients with altered sensorium. Finally, other limitations can be attributed to word selection used in the database key word search and the English language only inclusion criteria.

## 4. Discussion

Invasive interventions, like blind GT insertions, may be employed more frequently because of the increased complexity of health conditions seen with an aging healthcare population [64]. Its role as a vital treatment option for the management of patients requiring life-sustaining measures is well established. Moreover, its value in decompressing the stomach to prevent surgical injury remains a compelling argument for its continued use. It is the unsupported practices that are called into question.

Presently, evidence-based practice has decreased the utilization of GTs for postoperative decompression in abdominal surgical patients. The advent of new guidelines and pathway driven care has resulted in fewer morbidities and shorter hospitalizations [65] [66]. Several studies dating back to 1995 report the benefits of forgoing the traditional practices of postoperative gastric decompression for the management of laparotomy, gastrectomy and minimally invasive esophagectomy patients [67] [68] [69]. Its routine use has even been questioned in the management of small bowel obstructions [70]. Accordingly, anesthesia providers would benefit by developing specific practice guidelines based on current evidence. More importantly, based on the benefits of eliminating GTs as a modality for the treatment of postoperative bowel issues, anesthesia providers should advocate for the removal of pre- and intra-operatively placed GTs when clinically appropriate. Another issue that has not been addressed in literature is the advent of Enhanced Recovery after Surgery (ERAS) protocols that now call for preoperative dosing of NK-1 receptor antagonists and GABA-analog anticonvulsants. The placement of a GT near to the time of oral administration can effectively remove the unabsorbed medication and therefore should be taken into consideration before insertion.

Blind GT intubations have limited utility in elective scenarios and expose otherwise healthy patients to considerable harm. Therefore, more stringent criteria should be used when advocating its elective use in the intraoperative setting. It is the opinion of this reviews’ authors, that in the absence of aspiration risk factors or the need for surgical decompression in ASA classification I & II patients, a moratorium should be instituted on the elective use of GTs in these patients undergoing GETA.

## 5. Conclusions

This review actually raises more questions than it answers and is consequent to the paucity of anesthesia literature pertaining to this “other tube.” Gastric tubes used intraoperatively are not associated with a decrease in aspiration risk, only a decrease in aspirate content. Also, the majority of aspirations (68%) occur during induction and emergence [71]. It would not be conjecture to assume that more of the focus should be on anesthetic technique and management like optimal cricoid pressure and extubation after complete reversal from neuromuscular blockade, respectively.

The intent of this review is to offer a comprehensive exploration and evaluation of literature that can be correlated with anesthesia provider GT practice. Current published literature is severely deficient in the coherent compilation of information related to GT utilization by anesthesia providers. It also provides a more complete dissemination of new information that will greatly complement previous review articles communicating related literature. Notably, this review disseminates clinically relevant information that is applicable to a potentially harmful practice modality employed by anesthesia providers. Its attention to possible complications, malposition risk factors, verification methods, and correct placement facilitating schemes could and have provided the groundwork for guideline/pathway derivation that can be aligned with previously proposed algorithms.

This is especially important with the enactment of the Patient Protection and Affordable Care Act (PPACA) and the role it will play toward quality improvement. One such quality improvement provision involves the Centers for Medicare & Medicaid Services (CMS) and its reimbursement practices. The CMS will no longer reimburse hospitals for preventable readmissions, which they estimate costs the taxpayer billions of dollars [72]. The development of a practice guideline/pathway for the use of GT in patients, not requiring surgical gastric decompression, could be one such intervention that could prevent injuries and the associated iatrogenic high morbidity. The “do no harm” principle is the basis of an oath every healthcare provider assumes when accepting the responsibility and acting as an advocate. All healthcare providers should question practices that are ineffective or potentially harmful and support practices based on current evidence and less on “provider preference”.

## Figures and Tables

**Table 1:** Review of complications by case study.

Case Study	Demographics	Attempts	Complications	Outcomes
Hynh 2009	67 year-old male Esophagectomy	One	Gastric fistula	Emergent thoracotomy
Hirshoren 2009	77 year-old female Embolectomy	Multiple	Pharyngeal injury, infected hematoma	Irrigation and debridement of infected wound
Hegde 2010	60 year-old male Intensive care unit	Multiple	Bronchial malposition	Pneumonia, death (may not be related to gastric tube)
Khanna 2012	22 year-old male Gastric pull-up	One	Indeterminate position	No adverse sequel
Lin 2012	47 year-old male Peptic ulcer repair	Two	Endotracheal tube coiling and constriction	Pulmonary edema
Daliya 2012	32 year-old male Laparotomy	Not specified	Gastric perforation	Emergent laparotomy
Nanjegowda 2013	45 year-old male Cholecystectomy	One	Tracheal malposition	Recurrent laryngospasm
Ranier 2013	50 year-old Anterior/posterior spinal fusion	One	Fractured tip	Coughed up fractured tip in recovery room
Kerforne 2013	44 year-old female Gastric bypass	One	Tracheal malposition	Tracheal aspiration of dye
Turabi 2014	39 year-old female Shoulder surgery	Multiple	Esophageal perforation, pneumothorax	Esophageal stent, chest tube
Ching 2014	78 year-old male Esophagogastrectomy	One	Unsuccessful attempt	Surgical facilitation to prevent further injury
Govindarajuru 2014	60 year-old male Cholecystectomy	One	Tracheal malposition	Removed without sequel
Joseph 2014	66 year-old female Tracheostomy	One	Pulmonary malposition	Ventricular tachycardia, pleural effusion, pneumonia
Isik 2014	70 year-old male Cholecystectomy	Not specified	Esophageal perforation	Emergency thoracotomy, esophageal repair
Burad 2014	52 year-old female Aneurysm coiling	Two	Laryngeal injury	Esophagoscopy
Acharya 2014	60 year-old male Laparotomy	Two	Gastric tube knotting around endotracheal tube	Removed without sequel
Sahu 2015	31 year-old Cholecystectomy	One	Tracheal malposition	Removed without sequel
Bagharwal 2015	17 year-old female Gastric pull-up	Multiple	Gastric tube and nasopharyngeal temperature probe entanglement	Nasal bleeding
Kalava 2015	64 year-old male Mandibular surgery	One	Bronchial malposition	Removed without sequel in recovery room after x-ray revealed misplacement
Garg 2015	35 year-old female Cholecystectomy	One	Self knotting of gastric tube through supraglottic device	Removed objects en masse without sequel
Raut 2015	70 year-old male Coronary bypass & graft	One	Bronchial malposition	Removed without sequel in intensive care unit after x-ray revealed misplacement

## Abbreviations

ASA: American Society of Anesthesiologists
PONV: Postoperative Nausea and Vomiting
GETA: General Endotracheal Anesthesia
GT: Gastric Tube
ETT: Endotracheal Tube
EGD: Esophagogastroduodenoscopy
RCT: Randomized Controlled Trial
CO_2_: Carbon Dioxide
ERAS: Enhanced Recovery After Surgery
NK: Neurokinin
GABA: *gamma*-Aminobutyric acid
CMS: Centers for Medicare and Medicaid Services
PPACA: Patient Protection and Affordable Care Act
